# Parental participation and parents’ support: effects on mathematics achievement, 2018 national assessment of learning, Mexico

**DOI:** 10.3389/fpsyg.2023.1154470

**Published:** 2023-07-05

**Authors:** Eduardo Hernández-Padilla, Aldo Bazán-Ramírez, Wilfredo Bazán-Ramírez, Juan Solano-Gutierrez

**Affiliations:** ^1^Centro de Investigación Transdisciplinar en Psicología, Universidad Autónoma del Estado de Morelos, Cuernavaca, Morelos, Mexico; ^2^Departamento Académico de Educación y Humanidades, Universidad Nacional José María Arguedas, Andahuaylas, Peru; ^3^Facultad de Ciencias Administrativas, Universidad Nacional Mayor de San Marcos, Lima, Peru

**Keywords:** family support, math learning, socio-economic level, secondary education, academic achievement

## Abstract

The relationship between family involvement in students’ academic activities, their learning, and academic achievement has been widely studied. Two different types of family involvement are recognized: parental participation, which is linked to activities implemented by the school, and parental support, which occurs at home and has to do with the family’s educational practice. This study analyzed the influence of parental participation in school activities, parental support at home, and family socioeconomic status on student learning in mathematics. The database of 104,973 third-year secondary education students (50.1% female and 49.9% male) from the 2018 assessment of academic achievement in mathematical thinking was considered. Path analysis was employed in structural equation modeling, where a general model of parental support was proposed to compare the learning of students from the lowest quartile and those from the highest socioeconomic level; the model presented a good fit for each group. The models used showed acceptable adjustments in some criteria; in these models, there were positive and significant associations between parental schooling and socioeconomic level and achievement in mathematics. The results obtained are consistent with the findings of other studies in which parental support, mediated by socioeconomic status, significantly influences student learning.

## 1. Introduction

The interaction of school and family, as the first agents of socialization, significantly influences learning, academic achievement, and the quality of education that students receive. The frequency and quality of the relationships that can be established between both agents will determine, among other aspects, the student’s school performance. The relationships between academic centers and parents promote cooperative work between both, where the involvement of parents in the schoolchildren’s learning can promote the educational achievement of students (Xu et al., 2010; Álvarez, 2019).

The benefits of involving families in children’s education are among the most compelling and consistent findings in the educational literature. There is overwhelming research support attesting to the effects of parental involvement and family-school partnership practices in positively advancing children’s education Trajectories (Sheridan et al., 2016). Research trends suggest that family-based educational interventions appear to be most successful when (a) they involve collaboration between families and schools; (b) promote healthy relationships between families and schools, and parents and children; and (c) use evidence-based parent-teacher practices.

The importance of parental involvement in education is such that, in addition to being a critical indicator of educational quality, it is valued as an essential element to advance the fulfillment of educational effectiveness and improvement (Fan and Chen, 2001; Jeynes, 2005, 2007, 2012; Epstein, 2011). Therefore, schools, universities, and departments of education (SCDE) in the United States have focused on preparing educational leaders and graduates to carry out family and community engagement activities and generate innovations in the school-family relationship (Epstein and Sanders, 2006).

Parental involvement becomes especially relevant given the need for school autonomy with which it has a positive relationship, where greater autonomy would strengthen parental involvement to the point of constituting it as a constructive force that, in turn, will enhance the efforts of schools for the benefit of students (Grant and Ray, 2013), particularly their academic performance (Robinson and Harris, 2014). The interaction between school autonomy and parental support has greater relevance in vulnerable groups, such as ethnic minorities and students from low socioeconomic levels and disadvantaged demographic areas (Driessen et al., 2005). The educational effectiveness of parental support begins to manifest itself in the preschool period (Biedinger, 2010); this practice has not gone unnoticed by educational agents or by those who exercise policies concerning those involved in the teaching and learning processes (Wilder, 2014).

Despite the evidence highlighting the importance of parental involvement, as previously noted, the definition of the term has been ambiguously conceived and presents several inconsistencies (Fan and Chen, 2001; Durand, 2011; Bazán et al., 2014; Wilder, 2014; Miranda-Carvajal and Castillo-Armijo, 2018; Nygreen, 2019; Mayger and Provinzano, 2021). With the term family involvement, various actions on the part of parents have been characterized with respect to the academic development of their children and the relationship of the family with the school. This has made it possible for up to three general but interrelated terms to be used in the literature on the family school relationship: parental involvement, participation, and support. These terms are briefly discussed below.

### 1.1. Parental involvement and achievement

Research on the family school relationship with respect to student academic development, especially in the United States, has experienced a great boom since the research on parental involvement in children’s education (Epstein, 1995) and the significant effects of parental involvement on student academic development, especially in schools and school districts, with better implemented programs and actions (Akmal and Larsen, 2004).

The involvement of parents in student learning to generate a positive effect on student academic achievement was characterized by various actions that parents carried out to get involved in programs or activities promoted by schools and higher education authorities. Among these activities are parenting, learning at home, communication, volunteering, school decision-making, and collaboration with the school (Epstein, 1995, 2001, 2011; Epstein and Sheldon, 2006).

Fan and Chen (2001) conducted an early meta-analysis to synthesize the quantitative literature on the relationship between parental involvement and student academic achievement. The authors found significant but small-to-moderate relationships between family involvement and academic achievement and reported that parents’ expectations of their children’s academic achievement had the strongest relationship, while parental supervision of academic work at home had the weakest relationship with students’ academic achievement. These results also reflect the multidimensional nature of parental involvement.

Contemplated more broadly, parental involvement, can range from educational expectations, supervision, and accompaniment in the study of their children, the support they provide to them in what they learn at school and in extracurricular tasks, as well as the frequency in which they attend school, all with the specific objective of improving the academic performance of students (Robinson and Harris, 2014; Castro et al., 2015; Silinskas and Kikas, 2019).

Hong et al. (2010) found that two family involvement variables had different effects on student achievement in mathematics: parents’ valuing of mathematics had a significant effect, but parents’ academic reinforcement of their children had no significant effect. Family involvement, shaped by student-reported indicators of parental involvement in student learning, direct communication about student progress, and providing general information about school principles and activities, has a positive and significant effect on student achievement in mathematics (Shapira-Lishchinsky and Zavelevsky, 2020).

Based on several meta-analyzes on family involvement and academic performance, Jeynes has pointed out that, in the scientific literature, many more categories and terms describing various actions of family involvement have been included, which can be grouped into seven categories: specific parental involvement. Communication, Homework, Parental expectations, Reading, Attendance and participation and Parental Style (Jeynes, 2005, 2007, 2010, 2012). For the predictive analyzes between family involvement and academic performance, Jeynes maintained an index of the term general parental involvement, which is a variable composed of indices of the seven categories of family involvement.

A meta-analysis study in Asian countries showed a significant effect of three family involvement variables on academic achievement: academic socialization, home involvement, and school involvement (Kim, 2020). Similarly, in a recent systematic review on parental involvement and academic achievement in mathematics, the various involvement variables were grouped into five general categories: Learning, Belief, Motivation, Emotion, and Behavior, and showed that many of the involvement indicators are significantly related to academic achievement (Fiskerstran, 2022).

Despite the prolific field of studies on parental involvement and academic achievement, as previously noted in the introduction, there have been questions about the validity and usefulness of various indicators or variables of involvement. One example is Jeynes’s (2005, 2007, 2012) definition of parental involvement as parental participation in the educational processes and experiences of their children.

### 1.2. Parental participation and parental support

Large-scale evaluations of academic achievement have been considered context variables that influence academic achievement, two constructs: parental participation and parental support (Organization for Economic Co-operation and Development, 2013, 2017; Instituto Nacional para la Evaluación de la Educación-INEE, 2019). Coincidentally, Bazán et al. (2014) have distinguished the concept of parental support from the participation model of the family and conceived as part of a genuine educational practice of the family, and parental participation as a school-oriented practice.

In the case of Parental Involvement, parents participate in various activities promoted by schools. In this approach, parental involvement is framed as an educational policy issue (Kim, 2020) and is also framed within the activities included in the initial approaches to parental involvement (Epstein, 1995; Hara and Burke, 1998; Epstein, 2001; Akmal and Larsen, 2004). Thus, the participation models contemplated the participation of parents as part of school-centered processes (Nygreen, 2019; Mayger and Provinzano, 2021). In research on the subject, the term family participation is commonly used as a component of parental involvement (Epstein, 2001; Jeynes, 2005).

Additionally, the term parental support implies family support for the educational process of their children, with or without the need for the school to invite parents to support the students. Parental support is voluntary (Henderson and Mapp, 2002), spontaneous (Desforges and Abouchaar, 2003), and constitutes an educational practice for the family (Barber, 1988; Reglin, 2002; Bazán et al., 2007). Parental support can be considered as active support of parents or guardians in the emotional, social and academic development of their children; it even becomes a key reference for the Quality of Educational Systems (Colás-Bravo and Contreras-Rosado, 2013).

Other authors define support as activities at home aimed at improving children’s literary skills (Senechal and Young, 2008); homework assistance, or at least homework verification (Pezdek et al., 2002); parents’ help to children with homework (Patall et al., 2008); communication with children regarding their performance at school, participation in school activities in the school and home contexts, as well as expectations about educational achievement and parental attitudes toward education (Hill and Tyson, 2009).

In his meta-analysis, Jeynes (2012), found that there is a strong positive relationship between educational achievement and parental support, regardless of the definition of the latter; likewise, the results showed that the association is stronger if parental support expectations for their children’s academic achievement are included (Williams-Shanks et al., 2010; Long and Pang, 2016).

In parental support for learning at home, Latunde (2017) recognizes the existence of two types of parental involvement: a traditional and a heterodox one, both of which are effective in increasing student success. The traditional approach focuses on what parents can do for the school (e.g., homework support, help with educational expectations, volunteering, parent-teacher meetings, participation on school committees, and fundraising). Traditional types of involvement position schools, that determine the agenda and type of assistance, which will result in a lack of appreciation for other types of contributions in other groups such as marginalization.

Because of this, parents who come from a high socioeconomic and cultural level have a clear discernment of academic and extracurricular activities that can benefit the academic performance of their children, in addition to having high educational expectations of those with the consequent direction of considerable economic expenditure in educational activities and school participation (Kim et al., 2013); in contrast, parents of low socioeconomic level do not know how to support them (Bazán et al., 2014). In Mexico, it has been consistently shown that there is an association between the socioeconomic and cultural stratification of schools, due to the socioeconomic and cultural level of the parents who choose the services, and the marginalization index where the institutions are located, in such a way that In this way, it is possible to infer that where there is a higher concentration of socioeconomic and cultural level, there is also a higher concentration of income (Tapia and Valenti, 2016). These types of participation are those to which parents can resort because they often require minimal material resources, making them more accessible (Robinson and Harris, 2014). On the other hand, non-traditional forms of parental involvement are socially and culturally more inclusive, where home and school involvement is recognized, which employ modeling, motivation, communication of educational values, joint management, and other forms of support that are not dominant (Latunde, 2017); but which also demonstrate that parents’ willingness to participate in homework, their supervision, and help with homework at home is one of the highest forms of parental involvement (Thornton, 2015).

In their work, Bazán et al. (2016) also identified two constructs of parental activities related to the school: the first construct they called “Parental involvement at school” and includes the academic activities of school management, building maintenance, and supervision of the student’s education organized by the school or carried out on the parents’ initiative. The second factor or construct was called “Parental support for learning at home,” which was made up of the regularity with which parents or guardians carry out extracurricular activities with the student, such as helping in these activities, talking about a topic or subject, providing educational material, and communicating with the student. However, the literature reports the inclusion of both indicators of parental participation or involvement, as well as indicators of parental support, and their significant effects on academic performance in various contexts and educational levels (Bazán et al., 2007, 2016; Boonk et al., 2018; Lara and Saracostti, 2019; Murillo and Hernández-Castilla, 2020).

### 1.3. Socioeconomic and parents’ education level, and achievement in mathematics

In the existing literature on the factors associated with academic performance, socioeconomic factors are linked to cultural capital (Organization for Economic Co-operation and Development, 2016), the latter representing parental schooling and parental occupational status. Several studies have shown that both socioeconomic and parental education levels are significant predictors of academic achievement (Hernández and González, 2011; Reyes et al., 2014; Cuellar-Caicedo et al., 2016; Sánchez et al., 2016; Tristán, 2018; Tristán et al., 2019; Weis et al., 2022).

On the other hand, socioeconomic level is significantly associated with learning (Hernández and Bazán, 2016; Organization for Economic Co-operation and Development, 2016; Kaya and Erdem, 2021), and different studies have demonstrated the association between a positive relationship between parental schooling level and academic achievement (Said-Rücker, 2011; Organization for Economic Co-operation and Development, 2016; Silinskas and Kikas, 2019).

### 1.4. Current study

In Mexico, various studies have verified the effect of various context factors on academic performance in national and international tests. In these works, samples with a national representativeness have been used, employing analyzes such as multilevel hierarchical modeling or structural equation modeling. Among the different factors evaluated are the socioeconomic and cultural level of the family (Backhoff et al., 2007); the educational inequalities of the educational system (Blanco, 2017); the type of school (Moreno and Cortez-Soto, 2020); the type of service (Hernández-Padilla, 2018); including the impact of family support on learning (Bazán et al., 2016). Particularly this last topic has been little addressed in studies on academic performance in Mexico, and the results do not seem to be conclusive.

The primary goal of the current research was to identify the effect predictor of the direct relationships between parental socioeconomic and educational status and academic math achievement, as well as the relationship between these two factors and parental involvement. The relationship between parental support and math academic success is examined in Mexico’s 2018 National Plan for the Evaluation of Learning (PLANEA). Determining the degree of correlation between the two main predictor variables—the parents’ educational level and socioeconomic status—was a secondary goal.

The following questions served as a guide for the investigation of mathematical learning through structural equation modeling in order to take the goals of this study into consideration.

1. What is the relationship of parental socioeconomic status (household resources) and parental education level to parental involvement and support?
2. What are the effects of parental socioeconomic status (household resources), parental educational attainment, parental involvement, and parental support on the mathematics achievement of high school students?

## 2. Methods

### 2.1. Data and sample

The indicators of the suggested factors came from the context questionnaires of 104,973 third-grade Mexican high school students (females, 50.1%; and age mean = 12.03 and standard deviation = 0.51 years [the average age is lower than the expected 15 years due to the fact that in the Mexican educational system, approximately 20.4% of students in secondary education are ahead of their corresponding educational level by age (Instituto Nacional para la Evaluación de la Educación-INEE, 2019)], from 3,573 schools in the 32 states of the country, who participated in the National Plan for the Evaluation of Learning (Planea), in its modality of Evaluation of Achievement referred to the National Educational System (ELSEN) Mathematical Thinking 2018. The sample has representativeness at the national, state, and school-type levels. From the students’ context questionnaires, the following factors were obtained: Socioeconomic Level; Parental Schooling; Parental Support; and Mathematics. The factor of Parental Involvement by the teacher was obtained from the questionnaires applied to teachers.

### 2.2. Modeled variables

To evaluate the proposed theoretical model, a combination of latent variables (factors) and measures was used. The construction of each is described below.

#### 2.2.1. Socioeconomic level

This factor is made up of the total number of belongings at home: computer or laptop, plasma television, cell phones, tablets, and DVD or Blu-Ray players, and services such as pay television, and Internet; likewise, appliances such as stove or landline telephone (Cronbach’s Alpha = 0.747) which has been significantly associated with learning (Hernández and Bazán, 2016; Organization for Economic Co-operation and Development, 2016; Kaya and Erdem, 2021).

#### 2.2.2. Parents’ education level

Parental schooling was evaluated from the absence of formal studies to higher or postgraduate education, considering whether the educational level was completed or not (Pearson correlation = 0.547 *p* < 0.001). Different studies have demonstrated the association between a positive relationship between parental schooling level and academic achievement (Said-Rücker, 2011; Organization for Economic Co-operation and Development, 2016; Silinskas and Kikas, 2019).

#### 2.2.3. Parental participation in school activities

The collaboration of parents in various school activities such as attending information meetings on the academic progress of students, to the participation in furniture maintenance activities, are actions that allow the interaction of the family in the classroom with the teacher, where contributions to the implementation of the school curriculum are also exposed (Cronbach’s Alpha = 0.737), thereby increasing the likelihood of parental involvement at home (Hill and Tyson, 2009; Epstein, 2011; Murillo and Hernández-Castilla, 2020; Zhou et al., 2022).

#### 2.2.4. Parental support

Parental support in their children’s education is registered in the activities at home where they help the student in their extracurricular work, solve doubts, and establish communication with the student about what is happening in the teaching-learning process, motivate them to study, accompany and supervise the school work that their children do outside of school, etc. (Bazán et al., 2007, 2014, 2016; Latunde, 2017; Lara and Saracostti, 2019).

#### 2.2.5. Achievement in mathematics

The Planea 2018 test provides five “plausible value” scores per student to estimate significant learning in subjects such as Language and communication, and Mathematics, where the latter is recognized as “… a field that promotes problem-solving skills, the formulation of arguments to explain their results and the design of strategies and their processes for decision-making relies on reasoning rather than memorization” (Instituto Nacional para la Evaluación de la Educación-INEE (s/f), n.d.), through the use of multiple imputations to estimate individual latent achievement. The use of this method makes it possible to calculate more precise estimates, as well as to standardize the error term in large stratified samples (Wu, 2005).

Table 1 provides the means of student performance by the values of the variables that make up each factor. Not surprisingly, differences were found between the values of the variables, where the means of performance increase as the values of these variables increase. Students whose families have more assets and enjoy various services obtained higher values on average in Mathematics; on the other hand, the same occurs when students who respond that their parents help them with homework more frequently have higher achievement scores. Regarding the parents’ schooling, the differences between the extreme values, no schooling, and university degree, are up to one standard deviation (121 points) for fathers, while among women it is slightly lower (114 points). Finally, in the Teaching Participation factor, the parental activities reported by teachers show that the averages in educational achievement increase to the extent that such activities are more frequent.

**Table 1:** Sample frequencies and average of students in Planea 2018.

Secondary mathematics by socioeconomics level (the values of the answer choices are in parentheses)					
Socioeconomic level			% *N*	*μ*	(*σ*)
Stove	We have nowhere to cook	(0)	1.1	401	−0.37
	Firewood	(1)	11.5	459	−0.18
	Gas	(2)	79.1	513	−0.08
Light bulbs	No	(0)	39	498	−0.11
	Yes	(1)	61	506	−0.09
Landline telephone	No	(0)	22.5	490	−0.15
	Yes	(1)	77.5	510	−0.08
Internet	No	(0)	35.8	484	−0.11
	Yes	(1)	64.2	518	−0.09
Computers	None	(0)	44.5	484	−0.09
	1	(1)	33	512	−0.12
	2	(2)	13.4	542	−0.2
	3	(3)	5.2	544	−0.34
	4 or more	(4)	3.8	525	−0.4
Television	None	(0)	11.4	449	−0.17
	1	(1)	32.6	495	−0.12
	2	(2)	28	517	−0.13
	3	(3)	16.2	531	−0.18
	4 or more	(4)	11.8	538	−0.21
Automobile	None	(0)	36.2	488	−0.11
	1	(1)	34.3	507	−0.12
	2	(2)	17.1	524	−0.18
	3	(3)	7	508	−0.28
	4 or more	(4)	5.4	503	−0.3
Cell phone	None	(0)	14.2	456	−0.16
	1	(1)	19.3	474	−0.14
	2	(2)	13.4	507	−0.18
	3	(3)	16.4	525	−0.18
	4 or more	(4)	36.8	534	−0.12
Tablet	None	(0)	46.6	493	−0.1
	1	(1)	32.4	512	−0.12
	2	(2)	12.4	531	−0.21
	3	(3)	5	522	−0.33
	4 or more	(4)	3.6	504	−0.37
Blu-ray or DVD	None	(0)	32.4	486	−0.12
	1	(1)	42.6	515	−0.11
	2	(2)	15.6	530	−0.18
	3	(3)	5.3	524	−0.31
	4 or more	(4)	4.2	504	−0.35

Secondary mathematics by parental support and parental schooling (the values of the answer choices are in parentheses)					
Parental support			% *N*	*μ*	(*σ*)
Notebook revision	Never	(0)	17.4	495	−0.17
	Few times	(1)	37.1	507	−0.11
	Many times	(2)	23.6	514	−0.14
	Always	(3)	21.9	491	−0.14
Resolving doubts	Never	(0)	15.3	491	−0.18
	Few times	(1)	31.7	498	−0.12
	Many times	(2)	26.6	515	−0.14
	Always	(3)	26.5	505	−0.13
Ask what you have learned in school	Never	(0)	11.9	474	−0.19
	Few times	(1)	28.1	493	−0.13
	Many times	(2)	28.4	514	−0.13
	Always	(3)	31.6	513	−0.12
Help to study	Never	(0)	16.3	490	−0.19
	Few times	(1)	31.8	501	−0.12
	Many times	(2)	27.5	517	−0.14
	Always	(3)	24.4	512	−0.15
Resolving a doubt in school	Never	(0)	14.5	479	−0.18
	Few times	(1)	25.2	493	−0.14
	Many times	(2)	27.2	516	−0.14
	Always	(3)	33.1	521	−0.13
Parental schooling					
Father’s schooling	Do not know	(0)	20	505	−0.15
	Did not study	(1)	3.6	439	−0.29
	Incomplete primary	(2)	8.5	465	−0.21
	Complete primary	(3)	9.9	475	−0.2
	Incomplete high school	(4)	9	489	−0.2
	Complete high school	(5)	21.1	499	−0.14
	Preparatory, Baccalaureate or Technical career	(6)	15.4	521	−0.18
	University career or postgraduate (specialization, master’s degree or doctorate)	(7)	12.6	560	−0.21
Mother’s schooling	Do not know	(0)	13.2	499	−0.18
	Did not study	(1)	3.5	442	−0.32
	Incomplete primary	(2)	8.4	463	−0.2
	Complete primary	(3)	10.5	477	−0.19
	Incomplete high school	(4)	9.1	483	−0.21
	Complete high school	(5)	23.8	500	−0.13
	Preparatory, Baccalaureate or Technical career	(6)	17.6	526	−0.16
	University career or postgraduate (specialization, master’s degree or doctorate)	(7)	13.9	556	−0.2

Secondary mathematics by parental participation in school activities (the values of the answer choices are in parentheses)					
Parental participation in school activities			% *N*	*μ*	(*σ*)
Family ask for student progress	Never	(0)	4.1	490	−0.33
	Few times	(1)	63.7	496	−0.09
	Many times	(2)	25.1	523	−0.14
	Always	(3)	7.1	515	−0.28
Give suggestions to their children	Never	(0)	18	497	−0.16
	Few times	(1)	50	501	−0.1
	Many times	(2)	23.2	514	−0.14
	Always	(3)	8.8	506	−0.24
Repair furniture	Never	(0)	40.6	508	−0.11
	Few times	(1)	47.5	500	−0.1
	Many times	(2)	8.1	505	−0.24
	Always	(3)	3.8	507	−0.35
Provide materials	Never	(0)	25.2	502	−0.14
	Few times	(1)	52	500	−0.1
	Many times	(2)	16.4	513	−0.17
	Always	(3)	6.4	522	−0.28
Organize events	Never	(0)	21.4	503	−0.15
	Few times	(1)	54.6	500	−0.09
	Many times	(2)	17.6	513	−0.17
	Always	(3)	6.5	513	−0.28

Author’s estimates based on Planea 2018 secondary. Planea 2018 national test μ = 500 and σ = 100.

### 2.3. Structural modeling

**Figure 1:**
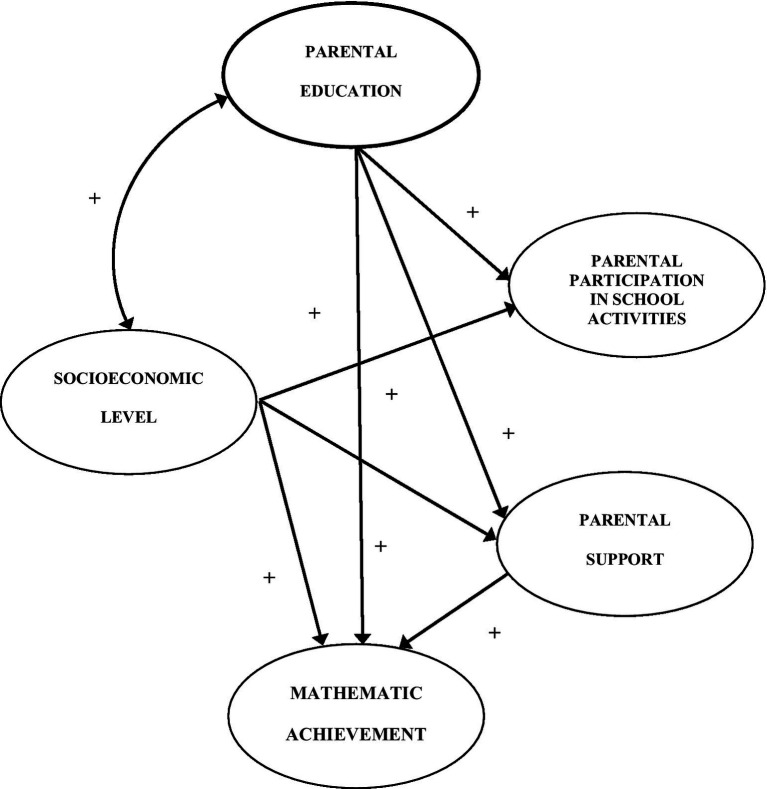
Theoretical model proposed for the analysis of parental participation and its interaction with other factors.

The general path model of structural equations that represents the hypothesis of the present work is shown in Figure 1. To have parsimony, only the factors of the model are shown in this figure; in this way, the direct effects to be estimated in Mathematics by the factors such as Teacher participation, Socioeconomic level, Parental schooling, and Parental support can be seen; as well as the influence of Parental schooling on Socioeconomic level, and the covariance of the former with PD. The EQS software (Bentler, 1995) was used to perform the above analysis; the models (overall, first quartile, and fourth quartile) were evaluated using three goodness-of-fit indices for their acceptance or rejection: the chi-square test, the comparative fit index (CFI), and the root mean square error of approximation (RMSEA). A non-significant value of the chi-square (i.e., *p* > 0.05) does not indicate rejection of the model, but its acceptance (although the significance of the model depends on the number of cases included in the modeling); on the other hand, the CFI values indicate that values ≥90 < 0.95 have an acceptable fit, while amounts above 0.95 indicate a very good fit; RMSEA values between ≤0.10 and > 0.05 are acceptable, and indices <0.05 indicate a good fit.

Due to the large number of the sample, criteria were used to measure the size of the effect and not its significance through the use of Cohen (1992) f 2 estimator to interpret partial or multiple correlations; f 2 values within an interval of 0.02–0.05 are considered “small”; “medium” values are between 0.06–0.09; finally, values equal to or greater than 0.10 are considered “large.” For the relationships between the different factors, positive coefficients or effects with Mathematics are proposed; likewise, the influence of those on parental support is also positive; the covariance between the educational level of the parents and parental support is also positive.

The interest of the present work is to know how these factors interact with Parental Involvement, which according to Latunde (2017), points out that orthodox parental practices, according to Epstein’s (2011) model, only favor students coming from high socioeconomic levels, while those coming from low strata have a negative association with academic performance (Latunde, 2017). For this purpose, a single measure of Socioeconomic level was performed using Rasch scaling employing the Winsteps^®^ program. The total sample was divided into four quartiles, where the first one represents the section of it that has fewer goods and services; while students located in the fourth quartile are those with a higher socioeconomic level. Table 2 shows the sample size used for each of the different models; the base model with which we worked is presented in Figure 1, the changes in the size of the effect of the factors and the disappearance of covariances were made under an empirical context and with the purpose of better fitting the model.

**Table 2:** Description of the models of “Direct and mediated effects of parental support, socioeconomic status, parental schooling, and parental participation in school activities on mathematics performance.”

Models	Description of the sample	*n*
M1	Third grade high school students who participated in the Planea 2018 sample	104,973
M2	Third-grade high school students belonging to the lowest quartile of socioeconomic level	21,284
M3	Third-grade high school students belonging to the highest quartile of socioeconomic level	28,825

Own elaboration based on information from the Planea 2018 Context Questionnaires for children and teachers.

## 3. Results

Table 3 shows the partial correlations (expressed in standardized values) between the factors. Statistically significant coefficients with manifest size effects (e.g., *f*^2^ ≥ 0.02; Cohen, 1992) are highlighted in the table.

**Table 3:** Coefficients and associated effect sizesa in the pathway models.

	General model		Model low socioeconomic level		Model high socioeconomic level	
*n*	104,753		21,284		28,825	
Path	r_syw_	*f* ^2^	r_syw_	*f* ^2^	r_syw_	*f* ^2^
EP — > Y	0.15	**0.02**	0.16	**0.03**	0.21	**0.05**
EP — > NSE	0.32	**0.11**	0.24	**0.06**	0.30	**0.10**
EP < —> PD	0.02	0.00	0.00	0.00	0.02	0.00
EP — > AP	0.17	**0.03**	0.17	**0.03**	0.19	**0.04**
NSE — > Y	0.20	**0.04**	−0.24	**0.06**	0.14	**0.02**
NSE — > AP	0.07	0.00	−0.04	0.00	0.07	0.00
PD — > Y	0.05	0.00	0.04	0.00	0.04	0.00
PD — > AP	0.01	0.00	0.02	0.00	0.00	0.00
PP — > Y	0.06	0.00	0.08	0.01	0.05	0.00
D_Y_ — > Y	0.95		0.96		0.97	
D_NSE_ — > NSE	0.95		0.97		0.95	
D_AP_ — > AP	0.98		0.99		0.98	

Effect size (*f*^2^ ≥ 0.02, Cohen, 1992); interpretation *f*^2^ = Small = 0.02–0.05; Medium = 0.06–0.09; Large = 0.10 or larger. Effect sizes of coefficients considered “notable” are highlighted in bold (see, *f*^2^ ≥ 0.02, Cohen, 1992). The interpretation of *f*^2^: Small = 0.02 to 0.05; Medium = 0.06 to 0.09; and, Large = 0.10 or greater. DY, DNSE and DAP, represent, in that order, the disturbances of Mathematics, Socioeconomic level, and Parental participation, the coefficients in bold are statistically significant.

To simplify the reading of the information, the error terms associated with each of the indicators of the factors that make up the models are eliminated. In the five plausible values of Mathematics, the factor loadings were found in a range of 0.93 to 0.96 across the three different models, with associated error terms ranging between 0.29 and 0.36. In the Parental Support factor across the different models, the associated error terms ranged from 0.70 to 0.86, while the loadings ranged from 0.59 to 0.71; in Parental participation in school activities, the loadings ranged from 0.48 to 0.71, while the errors ranged from 0.69 to 0.88. In other factors such as Socioeconomic Level, the errors are 0.0 to 1.0, while their coefficients ranged from −0.16 to 1.0. Finally, in Parental Education the errors range from 0.60 and 0.71, and their corresponding loadings from 0.70 to 0.80.

### 3.1. General model (M1)

**Figure 2:**
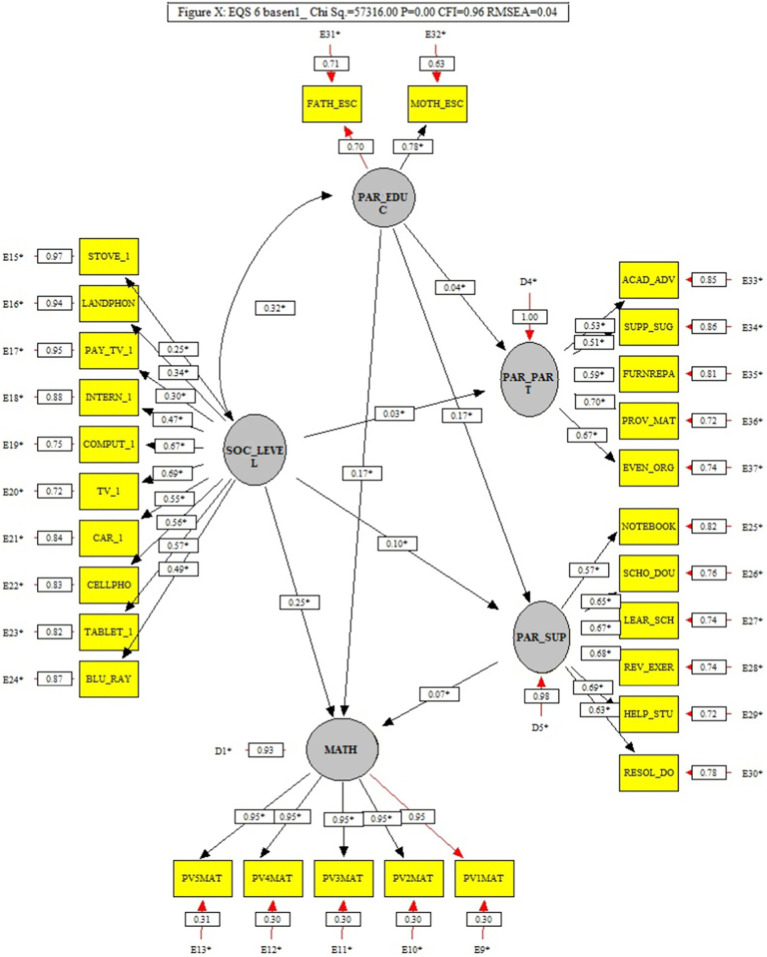
General model that analyzes the trajectories of the factors parental support and its interaction with parental schooling, socioeconomic level, and parental participation in school activities in mathematics.

The path analysis in the General Model, shown in Figure 2, showed a good fit (*x*^2^ = 57316.00, *p* > 0.05, CFI = 0.96, and RMSEA = 0.04), although, in it, Parental Support showed a low but significant association with achievement (0.07), and did not have a notable effect size (f ^2^ = 0.01). Similarly, of the other factors, only Parents education and Socioeconomic level had small effect sizes (*f*^2^ = 0.03 and *f*^2^ = 0.07, respectively) on achievement. On the other hand, parental education and socioeconomic level had a significant covariation (*f*^2^ = 0.11), while parental education had a small effect on parental support (*f*^2^ = 0.03).

### 3.2. Low socioeconomic level model (M2)

**Figure 3:**
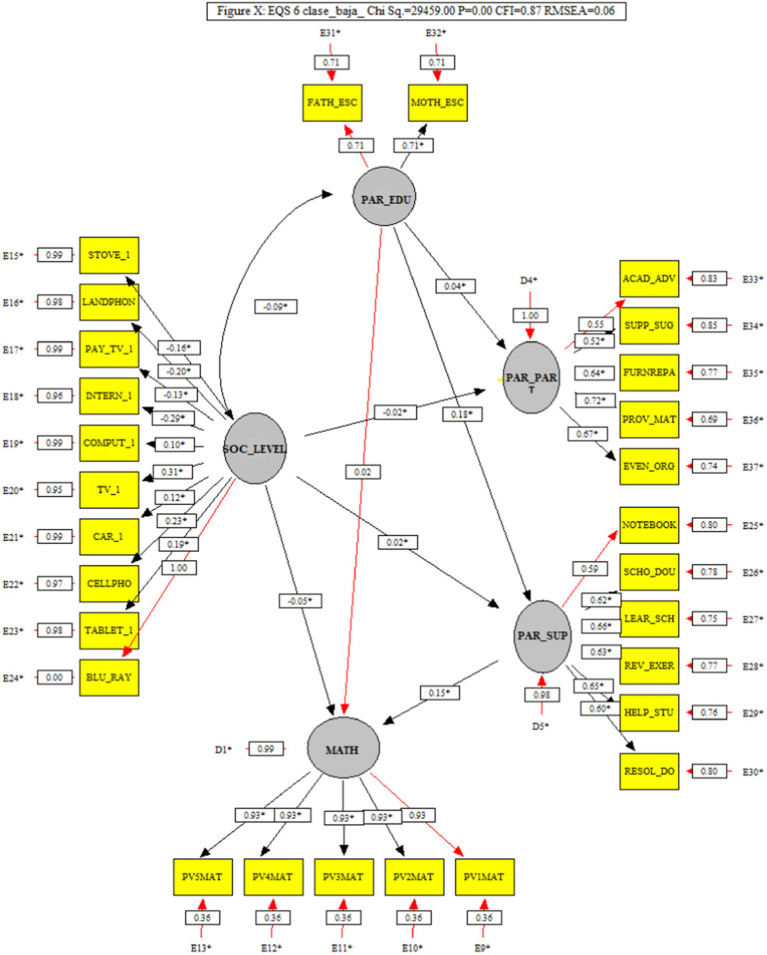
Low socioeconomic level model (M2) that analyzes the trajectories of the factors Parental support and its interaction with Parent schooling, Socioeconomic level, and Parental participation in school activities on mathematics.

The path analysis of this model can be seen in Figure 3, and shows that it also obtained indices that indicate a good fit (*x*^2^ = 29459.00, *p* > 0.05, CFI = 0.87, and RMSEA = 0.06), and as in the previous model, Parental Support showed low association with Mathematics (0.15), which is an effect of small size (*f*^2^ = 0.01).

Likewise, the factors Parent education (*f*^2^ = 0.02) and Socioeconomic level (*f*^2^ = 0.06), stood out for having the largest associations with achievement (Parent education = 0.16, and Socioeconomic level = − 0.24). In this model, Parent education showed a smaller relationship (0.24) with Socioeconomic level, compared to the previous model; while it maintained its influence on Parental support, although the effect is small (*f*^2^ = 0.03).

### 3.3. High socioeconomic level model (M3)

**Figure 4:**
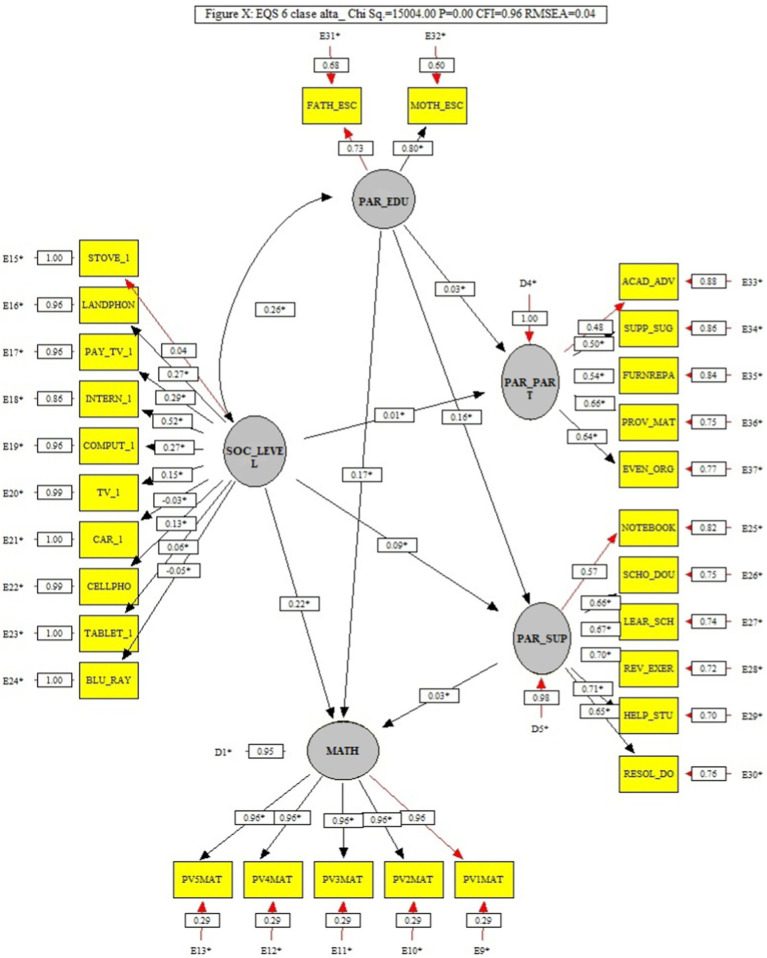
High socioeconomic level model (M3) that analyzes the trajectories of the factors of parental support and its interaction with parental schooling, socioeconomic level, and parental participation in school activities in Mathematics.

The last model (presented in Figure 4) also shows a good fit (*x*^2^ = 15004.00, *p* > 0.05, CFI = 0.96, and RMSEA = 0.04), where the association of Parental support with Mathematics achievement was smaller (0.03) than, in the previous models, and whose effect is not significant in size (*f*^2^ = 0.00). In this model, the factors that had the strongest association with Mathematics were Parents education (0.17 and *f*^2^ = 0.03), and Socioeconomic level (0.22 and *f*^2^ = 0.05). The magnitude of the relationship between Parents education and Socioeconomic level, in this model, obtained a value similar to that of the previous models (0.26), and with a large effect size (*f*^2^ = 0.07). Another relationship between factors that turned out to be low was that between parental education and parental participation in school activities (0.03), without a significant effect size (*f*^2^2 = 0.00).

## 4. Discussion

The importance of parental involvement in the education of their children has been studied in various works through which different types have been identified, mainly determined by the level of inclusion of parents in the educational process school (Kim et al., 2013), and/or by the assistance in school activities carried out at home, considered as traditional (Latunde, 2017).

About this last type of participation, called Parental Support in line with the work of Bazán et al. (2016), we estimated the influence that, together with other factors associated with it, it had on mathematics learning performance in the Planea 2018 test, employing path analysis in structural equation modeling. The three models employed showed acceptable fits in the CFI and RMSEA criteria, while *x*^2^ was statistically significant in each case, which can be attributed to the fact that this statistic is sensitive to sample size in structural equation modeling, meaning that when the values of n are large the probability that *x*^2^ is significant also grows (Bentler, 1995). In the general Model, positive and significant associations between Parental Schooling and Socioeconomic Level with Mathematics can be seen, which have notable size effects, which coincide with other works carried out (Hernández and González, 2011; Hernández and Bazán, 2016; Organization for Economic Co-operation and Development, 2016). On the other hand, although Parental support has a positive and significant association with achievement, the size of the effect is not notable according to Cohen’s (1992) criteria; the same as with Parental schooling, which does not coincide with the results of other authors who report that socioeconomic level is important because it is associated with how to support children (Bazán et al., 2014), and with the increase in assets for educational purposes (Robinson and Harris, 2014).

The relationships between factors and educational achievement presented in the general Model were also observed in the low and high socioeconomic level models both in size and significance; however, the relationship between the low socioeconomic level model, and the relationship of this factor with achievement, although statistically significant, is negative. This type of relationship has not been pointed out by previous works, which indicate a positive association between socioeconomic level and achievement (Hernández and González, 2011; Hernández and Bazán, 2016; Organization for Economic Co-operation and Development, 2016); this section of the sample may possess resilience skills or capacities that allow them to obtain good academic results despite their unfavorable socioeconomic and demographic conditions (Noriega et al., 2016). The Parental Support factor, in both socioeconomic models, shows to have a low positive and significant association with Mathematics, with very similar coefficients. The results obtained for the relationship between parental support and mathematics performance (0.05–0.08), coincide with the ranges, in standardized measures, by those reported by Jeynes (2005) and other authors (Bazán et al., 2016); thus, parental support, understood as the communication that is had with the student, the support or help in extracurricular work and the resolution of doubts in this regard, are positively associated with obtaining better learning results or academic achievement, which can be influenced by factors of Parents’ Schooling and Socioeconomic level.

One of the limitations of the present work is the construction of the factors that could be presented to the lack of technical and theoretical rigor of the context questionnaires (Jornet et al., 2012), where such factors were constructed with empirical criteria and not of a theoretical nature. The scope of parental involvement is broader and covers different activities and levels of parental involvement in their children’s education such as parental educational expectations among other indicators (Epstein, 2011; Bazán et al., 2016; Latunde, 2017). For example, in addition to the academic activities in which parents support their children, one could analyze the qualitative assessment that the latter have about parental support; At the same time, questions about parental support in ICT-related activities could be included. Similarly, measures of the family’s cultural capital can be included, since this variable has been included in other large-scale studies, such as the PISA tests, to explain academic performance.

To conclude this paper, it should be pointed out that in educational management at the federal, state, and municipal levels, it is not only important to make a greater effort in the economic support required by students to access the physical supplies and didactic materials that benefit them (Instituto Nacional para la Evaluación de la Educación-INEE (s/f), n.d.); but also to encourage the cooperation of parents or guardians in school activities, since they are educational agents capable of making effective contributions to student learning and school self-management (Colás-Bravo and Contreras-Rosado, 2013), which can counteract the deficiencies of educational environments, which would have a significant impact on student learning (Murillo and Hernández-Castilla, 2020). This is particularly true in those areas that traditionally show or exhibit deficits in educational material such as schools that have students with low socioeconomic status and little parental support (such as the results obtained in model 2). Similarly, as long as national and local education authorities do not allow parents’ social capital to access the school, students will not benefit from it; namely, the relatively high relationship between parental involvement may reflect the interests that parents have in the school that they wish to support physically and financially, which may be due to parents’ awareness of the role that the school plays in their children’s future life.

## Data availability statement

Publicly available datasets were analyzed in this study. This data can be found at: https://www.oecd.org/pisa/data/2018database/.

## Author contributions

EH-P elaborated and led the research project, led the data analysis, wrote the Spanish version of the article, and revised the English version of the manuscript. AB-R collaborated with the elaboration of the research project and data analysis, wrote the manuscript in Spanish, and revised the English version of the manuscript. WB-R collaborated with the elaboration of results, reviewed the Spanish version, and edited the English version of the manuscript. JS-G collaborated with the research and the elaboration of results and reviewed the English version of the manuscript. All authors contributed to the article and approved the submitted version.

## Conflict of interest

The authors declare that the research was conducted in the absence of any commercial or financial relationships that could be construed as a potential conflict of interest.

## Publisher’s note

All claims expressed in this article are solely those of the authors and do not necessarily represent those of their affiliated organizations, or those of the publisher, the editors and the reviewers. Any product that may be evaluated in this article, or claim that may be made by its manufacturer, is not guaranteed or endorsed by the publisher.
